# The impact of individualised cardiovascular disease (CVD) risk estimates and lifestyle advice on physical activity in individuals at high risk of CVD: a pilot 2 × 2 factorial understanding risk trial

**DOI:** 10.1186/1475-2840-7-21

**Published:** 2008-07-17

**Authors:** Hermione C Price, Lynne Tucker, Simon J Griffin, Rury R Holman

**Affiliations:** 1 Diabetes Trials Unit, Oxford Centre for Diabetes, Endocrinology and Metabolism, University of Oxford, Oxford, OX3 7LJ, UK; 2 MRC Epidemiology Unit, Institute of Metabolic Science, Box 285, Addenbrooke's Hospital, Hills Road, Cambridge, CB2 0QQ, UK

## Abstract

**Background:**

There is currently much interest in encouraging individuals to increase physical activity in order to reduce CVD risk. This study has been designed to determine if personalised CVD risk appreciation can increase physical activity in adults at high risk of CVD.

**Methods/Design:**

In a 2 × 2 factorial design participants are allocated at random to a personalised 10-year CVD risk estimate or numerical CVD risk factor values (systolic blood pressure, LDL cholesterol and fasting glucose) and, simultaneously, to receive a brief lifestyle advice intervention targeting physical activity, diet and smoking cessation or not. We aim to recruit 200 participants from Oxfordshire primary care practices. Eligibility criteria include adults age 40–70 years, estimated 10-year CVD risk ≥20%, ability to read and write English, no known CVD and no physical disability or other condition reducing the ability to walk. Primary outcome is physical activity measured by ActiGraph accelerometer with biochemical, anthropometrical and psychological measures as additional outcomes. Primary analysis is between group physical activity differences at one month powered to detect a difference of 30,000 total counts per day of physical activity between the groups. Additional analyses will seek to further elucidate the relationship between the provision of risk information, and intention to change behaviour and to determine the impact of both interventions on clinical and anthropometrical measures including fasting and 2 hour plasma glucose, fructosamine, serum cotinine, plasma vitamin C, body fat percentage and blood pressure.

**Discussion:**

This is a pilot trial seeking to demonstrate in a real world setting, proof of principal that provision of personalised risk information can contribute to behaviour changes aimed at reducing CVD risk. This study will increase our understanding of the links between the provision of risk information and behaviour change and if successful, could be used in clinical practice with little or no modification.

## Background

Assessment of physical activity is a key element of CVD risk evaluation [1]. A lack of regular physical activity may contribute to the early onset and progression of cardiovascular disease (CVD) in all individuals and almost any increase in physical activity is likely to result in health benefits. Physical activity has a wide variety of beneficial effects on the course of atherosclerosis, resulting in a 20–25% reduction in overall mortality[2].

For individuals with type 2 diabetes CVD risk is an immense problem as they have a 2–4 times greater CVD risk compared with non-diabetic individuals, even after adjustment for age, ethnicity, income, cholesterol level, systolic blood pressure and smoking[3]. Importantly, it is now recognised that increased risk of CVD is apparent long before the diagnosis of type 2 diabetes is made [4-6]. Physical activity reduces CVD risk and its impact is greatest in those at highest risk[7]. This is seen even when adjusting for weight loss indicating that physical activity is an independent risk factor for CVD and can provide some protection in those at highest risk[7,8]. In a meta-analysis of trials to promote physical activity among people with type 2 diabetes a 0.6% reduction in HbA1c was seen[9].

Individuals at risk of developing type 2 diabetes also benefit from physical activity as lifestyle interventions including physical activity have been shown to prevent or delay the onset of type 2 diabetes [10-12].

The beneficial effects of physical activity on CVD risk are mediated through a lowering of blood pressure, increasing HDL cholesterol, reducing body fat and reducing insulin resistance[7]. To date trials evaluating behaviour change interventions have been limited by a lack of use of objective measures of physical activity favouring imprecise self-report methods[13].

The study, conducted in individuals at high estimated CVD risk, aims to determine if the provision of individualised information about future CVD risk can be used successfully to change behaviour. Research from other fields indicates that interventions including an individualised risk estimate can have an impact on behaviour[14], particularly in those whose risk is higher than average for their age and sex[15].

Brief lifestyle interventions have been shown to be effective in preventing and treating type 2 diabetes[16] and in preventing the complications of diabetes including reducing CVD risk[17,18]. We expect to find that providing personalised CVD risk information or lifestyle advice to individuals at increased risk of CVD can increase the amount of physical activity they perform per day, compared with risk factor information alone or no lifestyle information. In turn, we expect also to see improvement in the levels of the other CVD risk factors measured.

## Methods/Design

### Primary objective

To determine the efficacy of each intervention for increasing physical activity at one month compared with CVD risk factor values and to determine if brief lifestyle advice versus no lifestyle advice can increase physical activity at one month.

### Secondary Objectives

To determine the efficacy of each intervention in modifying the following CVD risk factors: smoking status, fruit and vegetable intake, alcohol consumption, fasting and 2-hour post-challenge plasma glucose, fructosamine, waist circumference, weight, BMI, total cholesterol, HDL cholesterol, LDL cholesterol and triglycerides, systolic and diastolic blood pressure, estimated 10-year CVD risk, estimated risk of diabetes and body fat percentage.

### Tertiary Objectives

To determine the efficacy of each intervention on anxiety, quality of life, self-regulation, worry about future risk of heart attacks, intention to increase physical activity and recall of risk information given.

## Methods/design

### Study Design

**Figure 1:**
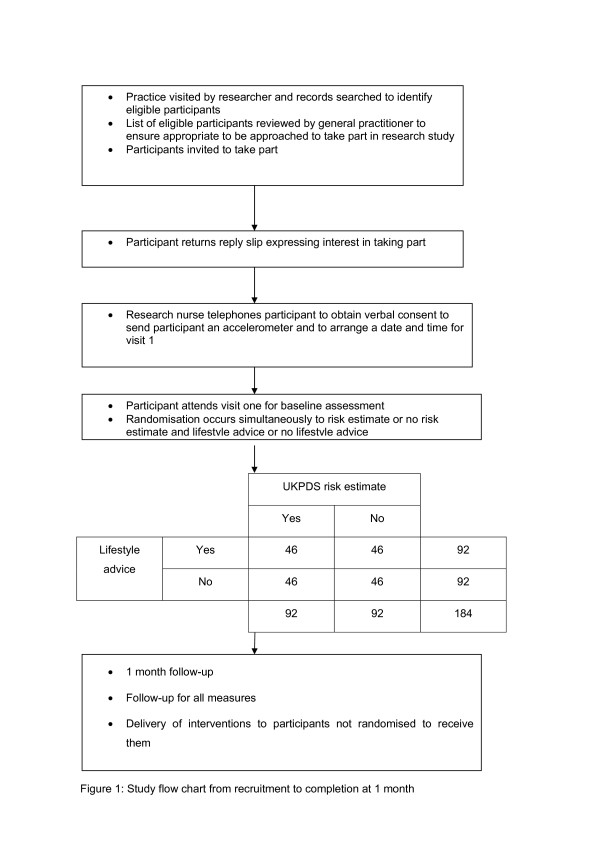


This is a pilot study to investigate the feasibility of a larger scale randomised controlled trial and is a 2 × 2 factorial design with 46 participants in each cell (Figure 1). This is considered to be the most appropriate and efficient method of answering the research question posed. Each participant will receive an individualised cardiovascular disease risk estimate or CVD risk factor values plus standardised brief lifestyle advice or not. The trial is being managed by a UK Clinical Research Collaboration Clinical Trials Unit, the Diabetes Trials Unit, University of Oxford, UK. Ethical approval was granted by the Milton Keynes Local Research Ethics Committee in September 2007.

Physical activity, clinical and psychological information will be collected before randomisation and one month after delivery of the intervention. A personalised CVD risk estimate and lifestyle advice will be provided to all participants at the end of 4 weeks making it a waiting-list control design. The inclusion of a control group receiving neither a risk estimate or lifestyle advice but undergoing baseline and follow up measurement will control for Hawthorne effects[19].

### Practices and participants

GP practices from Oxfordshire have been recruited. Participant eligibility criteria are adults aged 40–70 years, estimated 10-year CVD risk ≥20%, not known to have cardiovascular disease as defined by a history of myocardial infarction, other incident ischaemic heart disease, stroke, coronary or peripheral revascularisation, or transient ischaemic attack, not known to have a physical disability or other condition reducing the ability to walk, and able to read and write English.

### Screening eligibility

Participating practices allowed a member of the research team who holds an honorary contract with the Primary Care Trust, to identify eligible participants from practice databases. Once identified, lists of potentially eligible participants were constructed and general practitioners asked to review the list for suitability prior to sending out invitations to take part in the study. Participant invitation letters were sent out randomly in batches of 60 (figure 1).

### Telephone contact

Once a participant has expressed interest in taking part in the study by returning a reply slip in a prepaid envelope or by emailing or telephoning the research team a researcher obtains verbal consent and arranges a date and time for visit 1. ActiGraph accelerometers (model GT1M, Manufacturing Technology, Fort Walton Beach, FL) are then mailed to recruited participants approximately one week prior to visit 1 in order to reduce the number of visits and to reduce the potential effect of measurement on baseline physical activity. Simple instructions on how to use the device are provided and participants are asked to wear the accelerometer for seven days in total over a mixture of week and weekend days. Accelerometers are worn on an elastic belt around the waist and are removed for sleep, bathing, showering and swimming.

### Baseline assessment

Baseline clinical assessment is conducted by a clinical researcher and research nurses prior to randomisation in a clinical research unit. Written informed consent is collected at this visit. Medical history, demographic details, age at leaving school and electoral ward are collected. A 75 mg anhydrous oral glucose tolerance test (0 and 120 minute glucose sampling) is conducted and fasting blood samples taken for total, LDL and HDL cholesterol, triglycerides, HbA1c, serum cotinine, plasma vitamin C and fructosamine assessment. Anthropometric measurements are taken (resting blood pressure, height, weight, waist circumference, body fat percentage). Smoking status, current medication and self-reported alcohol intake is recorded. An ECG is performed. A booklet of questionnaires comprising the short-form Spielberger anxiety inventory[20], EQ5D questionnaire[21], Illness perception questionnaire, adapted Lerman cancer worry scale[22], psychological impact questionnaire, Recent Physical Activity Questionnaire (RPAQ) and A Fear-Avoidance Beliefs Questionnaire (FAB) is completed by each participant. Once these data have been collected the participant is randomised and any interventions delivered at this study visit.

### Outcome assessment

Follow-up at four weeks occurs for all measures including oral glucose tolerance test, blood samples, anthropometric measurements, smoking status, current medication, alcohol intake and questionnaires with the addition of a recall of risk information questionnaire. Physical activity is again measured by accelerometer for seven days in the week before the second study visit.

### Randomisation

Computer randomisation is used to allocate participants to each of the interventions in blocks of four. No minimisation is used. Research nurses who input data are blind to intervention allocation. One research fellow will remain unblinded in order to deliver the interventions. All research nurses working on the study have undergone training in the protocol and electronic data capture system prior to the study commencing.

### The interventions

Participants randomised to receive a risk estimate will have their 10-year CVD and fatal CVD risk estimated using a modified version of the UKPDS Risk Engine[23]. This has an interface designed specifically for use in the study with the aim of achieving maximum comprehension by participants. Participants will be shown their current risk and also their 'achievable risk' i.e. the risk estimate they could achieve if they were to meet current targets[24] for systolic blood pressure, LDL cholesterol, HbA1c and smoking cessation (if applicable). Participants will be given a UKPDS Risk Engine print out showing their current and achievable risk. Those participants not randomised to a 10-year CVD risk estimate will be informed of their LDL cholesterol, fasting plasma glucose and blood pressure values and informed if they are elevated according to current guidelines[24].

The lifestyle intervention used is based on self-regulation theory [25] and involves goal setting and steering behaviour towards goal achievement[26]. It also acknowledges the other priorities in participant's lives including family and financial constraints and encourages them to set goals around these commitments and states that any change in a positive direction is beneficial. This theoretical approach has been used in other studies attempting to motivate behaviour change in a similar population [27]. Participants randomised to receive the lifestyle advice intervention will receive a computer presentation. This presentation will focus on encouraging participants to increase their physical activity and fruit and vegetable intake and to decrease their alcohol intake and stop smoking if necessary. The physical activity goal will be 30 minutes of brisk walking per day in line with current guidelines[28]. Instant feedback of physical activity for example by using pedometers, is not provided as part of the study. The fruit and vegetable goal will be five portions per day in line with current UK government recommendations [29]. A reduction in alcohol consumption will only be advised if current intake is above UK government recommendations (not more than 3–4 units per day for men and 2–3 units per day for women)[30].

### Trial Management

The day-to-day management of the study will be undertaken by a clinical research fellow. Support will be provided by the co-investigators who will meet on an at least quarterly basis to discuss any matters that arise will the trial. The intervention is low risk and as such there will be no data monitoring committee. As this is not a clinical trial of an investigational medicinal product (CTIMP), there is no requirement for Medicines and Healthcare products Regulatory Agency (MHRA) approval or trial registration.

### Quality assurance

The Study will be conducted by trained personnel following departmental Standard Operating Procedures with Quality Control. Standardised delivery of the interventions will be assured by all interventions being delivered by one clinical research fellow using a computerised intervention. Source data verification will be performed by monitors from the University of Oxford Clinical Trials and Research Governance department.

### Sample size and rate of recruitment

The study has 80% power at the 5% level of significance to detect a difference of 30,000 total accelerometer counts per day of physical activity between the groups with a sample size of 46 per cell, assuming the estimated total counts per day is mean (SD) 275,400 (101,600) minutes[31]. This equates to 92 participants per arm of the study. We will randomise a total of 200 subjects to allow for a small drop-out rate. We expect retention of study participants to be high because of the short duration and non-invasiveness of the study. Recruitment is anticipated to continue for one year.

### Statistical analysis

Only data for participants who record at least 500 min/day of activity for at least 4 days during each 7 day period will be analysed. Data will be presented in terms of total body movement (counts·day^-1^), which is an indicator of the total volume of physical activity, and time (minutes·day^-1^) spent at different activity intensity categories averaged per day over the measurement period. Intensity thresholds for sedentary, light, moderate, vigorous and very vigorous intensity physical activity will be defined according to Freedson *et al *and Ekelund *et al*[32,33]. We will also determine the average number of 5- and 10-min bouts of sustained physical activity at moderate or greater intensity allowing 1 min to drop below the threshold for moderate activity in each 5-min bout. We will calculate the proportion of participants in each group accumulating 30 min or more per day of moderate or greater intensity physical activity, the current UK recommendation for physical activity[28]. Data reduction, cleaning, and analyses of accelerometer data will be performed using appropriate software (MAHUffe; see ). Generalized linear model (Poisson distribution or Negative Binomial distribution) as appropriate will be used and estimates of Rate Ratios along with 95% confidence interval will be presented.

Demographic, clinical and biochemical study variables will be summarized using number (percentage) and measures of location and dispersion as appropriate. The change in the study parameters from baseline to one month will be analysed and inference drawn using appropriate statistical methods, keeping in mind the nature of distributions of the study variables. Analysis will be by intention to treat (ITT).

To analyse the highly skewed EQ-5D scores, appropriate non-parametric technique will be used. Other questionnaire data will be analysed using appropriate non-parametric tests to compare average scores as well as distribution of scores in each group. Should a questionnaire generate only a finite number of scores, the data will then be considered polytomous and suitable statistical techniques will be used to analyse any such data.

Any missing biochemical data will be imputed using the Last Observation Carried Forward technique. Any missing accelerometer data however, will not be imputed for.

### Publication of results

It is anticipated that the results of the study will be published towards the end of 2009.

## Discussion

The aim of this study is to evaluate whether provision of personalised 10-year CVD risk estimates can increase intention to change behaviour and increase physical activity in individuals at high risk of CVD. The simultaneous randomisation to a risk estimate or not and to a lifestyle advice intervention or not will allow us to determine whether 10-year CVD risk information can increase intention to change behaviour and also if a brief lifestyle advice intervention can increase intention to change behaviour. The evidence that increased physical activity can reduce CVD is well established but our study represents a novel approach at trying to increase physical activity in a high risk group.

The outcome measures used have been chosen to provide objective measures of behaviour without undue inconvenience to participants. Accelerometers have been chosen as a measure of physical activity because they allow measurement of free living physical activity, avoid the need for whole-room calorimetry and are more accurate than the completion of physical activity questionnaires. 100,000 accelerometer counts are approximately equivalent to 30 minutes of brisk walking in a person of average size. In the MRC PROACTIVE trial, a behavioural intervention to promote physical activity among the 20–50 year old offspring of people with type 2 diabetes, the cohort exhibited a 10,000 increase in total counts per day between baseline (mean counts per day 264,000) and follow-up at one year (mean counts per day 274,000)[31]. Plasma vitamin C provides a robust measure of fruit and vegetable intake and has been shown to be inversely related to mortality from cardiovascular disease and ischaemic heart disease in men and women[34]. Serum cotinine is a reliable objective indicator of the quantity of tobacco smoked [35].

The Illness Perception Questionnaire has been included to provide insight into the influence of self-regulation on the decision to adhere to medical advice [36].

The study also includes measurements of potential mediators and moderators. Personality traits in particular optimism and pessimism (future orientation), are known to influence response to health information[37] this is measured by questionnaire[38] in order to examine its effect on the results of the study. Deprivation and a feeling of being unsafe in a community have been shown to be associated with lower rates of physical activity[29]. We collect age at leaving school and electoral ward as surrogate measures of deprivation. In order to investigate the impact of considering oneself already active on the results of the study we are using a recent physical activity questionnaire[39]. An adapted Lerman Cancer Worry Scale is being used to assess the impact of CVD risk information on worry about diabetes[22] although work in the field of pre-natal genetic screening suggests that numerical risk information can be successfully communicated to individuals and increase awareness of risk without raising anxiety[40].

Studies to investigate the use of personalised risk information are needed across a wide range of clinical topics to explore the impact of individualised risk estimates on knowledge, attitude, behaviour and patient understanding[41] with the aim of demonstrating a link between informed decision making and behaviour change[42]. We hope to demonstrate proof of principal that personalised risk information and brief theory-based advice can provide motivation to initiate behaviour change and to examine the intermediate measures that link changing beliefs to subsequent behaviour change. We have included a robust measure of physical activity and our intervention focuses on increasing activity as part of day to day life, factors that have limited the interpretation and applicability of other studies[13].

This project, if successful, has a high probability of providing generalisable results. Personalised risk scores can readily be assessed in many clinical situations and the intervention tested could be used in almost all individuals without modification.

## Funding

This trial is funded by an Insulin Dependent Diabetes Trust grant. HCP holds a Diabetes Trials Unit Fellowship.

## Competing interests

The authors declare that they have no competing interests.

## Authors' contributions

HCP and RRH conceived the study. All authors contributed to the design of the protocol and interventions. HCP drafted the manuscript. HCP will be responsible for the day to day management of the study overseen by LT, RRH and SJG.

## Investigators and trial steering group

Members of the writing group for this manuscript were HCP, LT, SJG and RRH.

The investigators are HCP, SJG and RRH.
